# Response to letter: Defining the optimal target for endovascular flow diversion using intracranial aneurysm and parent vessel morphometry

**DOI:** 10.1007/s00701-014-2246-y

**Published:** 2014-09-30

**Authors:** Tomasz Tykocki, Bogusław Kostkiewicz

**Affiliations:** 1 Department of Neurosurgery, Institute of Psychiatry and Neurology, Sobieskiego Street, Warsaw, 02-957 Poland; 2 Department of Neurosurgery, Central Clinical Hospital Ministry of Interior in Warsaw, Warsaw, Poland

Dear Editor,

We would like to thank Dale Ding for his comments on our manuscript ‘*Aneurysms of the anterior and posterior cerebral circulation: comparison of the morphometric features*’. Author referred to our study in the context of defining optimal criteria for the application of flow diverters, especially including morphometry of the intracranial aneurysms (IAs). Author’s doubts, regarding clinical transfer and utilization of our results in the decision-making process are somehow justified. However, first, we would like to emphasize that the scope of the study was to evaluate the potential morphometric differencies between IAs located in anterior or posterior cerebral circulation, and second, to define dissimilarities between ruptured and unruptured IAs. We did not investigate any issue dealt with the optimal treatment strategy (coiling, flow diversion, clipping etc.) with respect to aneurysm morphometry or location. The consistent and logical conclusion from our study is that IAs in anterior and posterior circulation have significantely different morphometric features (size ratio, aspect ratio, parent artery diameter). This might elucidate the well know fact why posterior circulation IAs have higer risk of rupture. Therefore, drawing any far-reaching conclusions about morphometry and flow diverters, although interesting, is an individual intention of the author.

Actually, in our opinion, there is not enough data to unambiguously recommend optimal target for endovascular flow diversion based only on the aneurysm morphometry. However, two issues should be taken into account during qualifying for flow diversion procedure, first, the characteristics of the aneurysm dome, and second, the morphology of the parent vessel itself. The assumption that flow diverters should be considered as an alternative (not a first choice treatment) mainly for IAs excluded from the treatment by other methods, especially by clipping or coiling may be correct. The detailed evaluation of the morphology of parent artery is particularly important from a technical point of view and should consider the tortuosity of the vessel, geometry of bi- or trifurcation, location of perforating branches and aneurysm inflow angle.

Finally, referring to the main hypothesis, we believe that in the light of current data it’s not possible to unequivocally define the optimal morphometric criteria for the use of flow diverters.

